# Combined shRNA over CRISPR/cas9 as a methodology to detect off-target effects and a potential compensatory mechanism

**DOI:** 10.1038/s41598-017-18551-z

**Published:** 2018-01-08

**Authors:** Liat Peretz, Elazar Besser, Renana Hajbi, Natania Casden, Dan Ziv, Nechama Kronenberg, Liat Ben Gigi, Sahar Sweetat, Saleh Khawaled, Rami Aqeilan, Oded Behar

**Affiliations:** 1 Department of Developmental Biology and Cancer Research, The Institute for Medical Research, Faculty of Medicine, The Hebrew University, Ein Kerem, P.O. Box 12271, Jerusalem, 91120 Israel; 2 Lautenberg Center for Immunology and Cancer Research, The Institute for Medical Research, Faculty of Medicine, The Hebrew University, Ein Kerem, P.O. Box 12271, Jerusalem, 91120 Israel

## Abstract

Inhibition of genes is a powerful approach to study their function. While RNA interference is a widely used method to achieve this goal, mounting evidence indicates that such an approach is prone to off-target effects. An alternative approach to gene function inhibition is genetic mutation, such as the CRISPR/cas9 method. A recent report, however, demonstrated that genetic mutation and inhibition of gene expression do not always give corresponding results. This can be explained by off-target effects, but it was recently shown, at least in one case, that these differences are the result of a compensatory mechanism induced only by genetic mutation. We present here a combination of RNA inhibition and CRISPR/cas9 methods to identify possible off targets as well as potential compensatory effects. This approach is demonstrated by testing a possible role for Sema4B in glioma biology, in which our results implicate Sema4B as having a critical function. In stark contrast, by using shRNA over CRISPR/cas9 combined methodology, we clearly demonstrate that the Sema4B targeted shRNA effects on cell proliferation is the result of off-target effects. Nevertheless, it also revealed that certain splice variants of Sema4B are important for the ability of glioma cells to grow as individual clones.

## Introduction

Small interfering RNA (siRNA) is widely used as a powerful tool for studying loss-of-function phenotypes in mammalian cells. One of the apparent advantages of using siRNA is its ability to silence genes in a sequence-specific manner. Indeed, a resource such as the Mission shRNA library provided by the RNAi Consortium (TRC) offers a convenient and affordable way to study loss-of-function of any human or mouse genes. However, a growing body of evidence suggests that siRNA specificity is not absolute and off-target gene silencing can occur through different mechanisms^1^. In attempt to address this problem, a number of approaches have been published, such as an introduction of random nucleotides into the guide strand to mitigate the off target effects, structurally asymmetric siRNA targeting, or reduced concentrations based on individual potency^2–4^. In addition, it is generally assumed that consistent results achieved by a few different siRNAs targeting different sequences in a specific gene alleviate this problem. Lastly, rescue experiments are a good way to ensure specificity and are being added to an increasing number of studies, although, based on a survey of scientific literature, this is probably limited to less than 0.1% of studies. The discovery of the CRISPR-Cas9 system as an efficient way to manipulate gene expression and function by genome engineering offers an alternative approach to studying loss-of-function phenotypes^5^. Recent comparisons between the two methods indicate that at least for some biological questions, the CRISPR-Cas9 system may be superior^6,7^. However, this approach also relies on relatively short sequence-specific recognition, and might therefore also be impacted by off-target effects, as has also been reported^8^. An additional problem that might influence the interpretation of loss-of-function approaches using this system is the possibility of compensation. Accumulating reports revealed phenotypic differences between knockouts (mutants) and knockdowns (RNA inhibition) in different model organisms including mouse, zebrafish and human cell lines^9–14^. These phenotypic differences may be the result of toxicity or off-target effects of the knockdown reagents. However, it is apparent that not all differences detected can be attributed to off-target effects of the anti-sense approach. In the case of the egfl7 gene, anti-sense morpholino exhibited a severe vascular defect, while genetic mutation of this gene had no phenotype^15^. Nevertheless, it was shown that the lack of phenotype in the case of the genetic mutation is the result of a compensatory mechanism. In contrast, this compensatory mechanism was not achieved by anti-sense inhibition, possibly because repression of the gene function is more modest or perhaps because the genomic lesions themselves might trigger a change upstream of the mutated gene^14,16^. Thus, when comparing RNA inhibition to genomic mutations, one should consider that complete loss of function by genetic mutants may induce a compensatory response, while RNA inhibition may generate off-target effects. Here, we present the case of Sema4B as a possible regulator in glioma biology and demonstrate an approach to differentiate between compensatory mechanisms and off-target effects using combined shRNA over CRISPR-Cas9 methodology.

The CNS tumor classification of the World Health Organization (WHO) recognizes a multitude of different neoplastic CNS entities, of which malignant gliomas (glioblastomamultiforme, GBM) are the most common primary malignancies. GBMs are characterized by necrotic, hypoxic areas and a prominent, proliferative vascular component. While looking for new genes involved in glioma tumorigenic phenotype we decided to test one of the members of the semaphorin family, namely Sema4B. Sema4B, a type 4 semaphorin, is a transmembrane protein with a short intracellular domain. Sema4B has been implicated in both tumor invasion and proliferation, mostly in lung cancer cells^17–19^. A possible role of this protein in glioma, however, has not been tested. We have recently shown that Sema4B has a role in astrocyte (a type of glial cell) proliferation and therefore decided to test whether this protein has a function in glioma formation^20^.

## Results

### Sema4B is expressed in glioma cell lines and knockdown of this gene reduces proliferation and increases cell death

**Figure 1:**
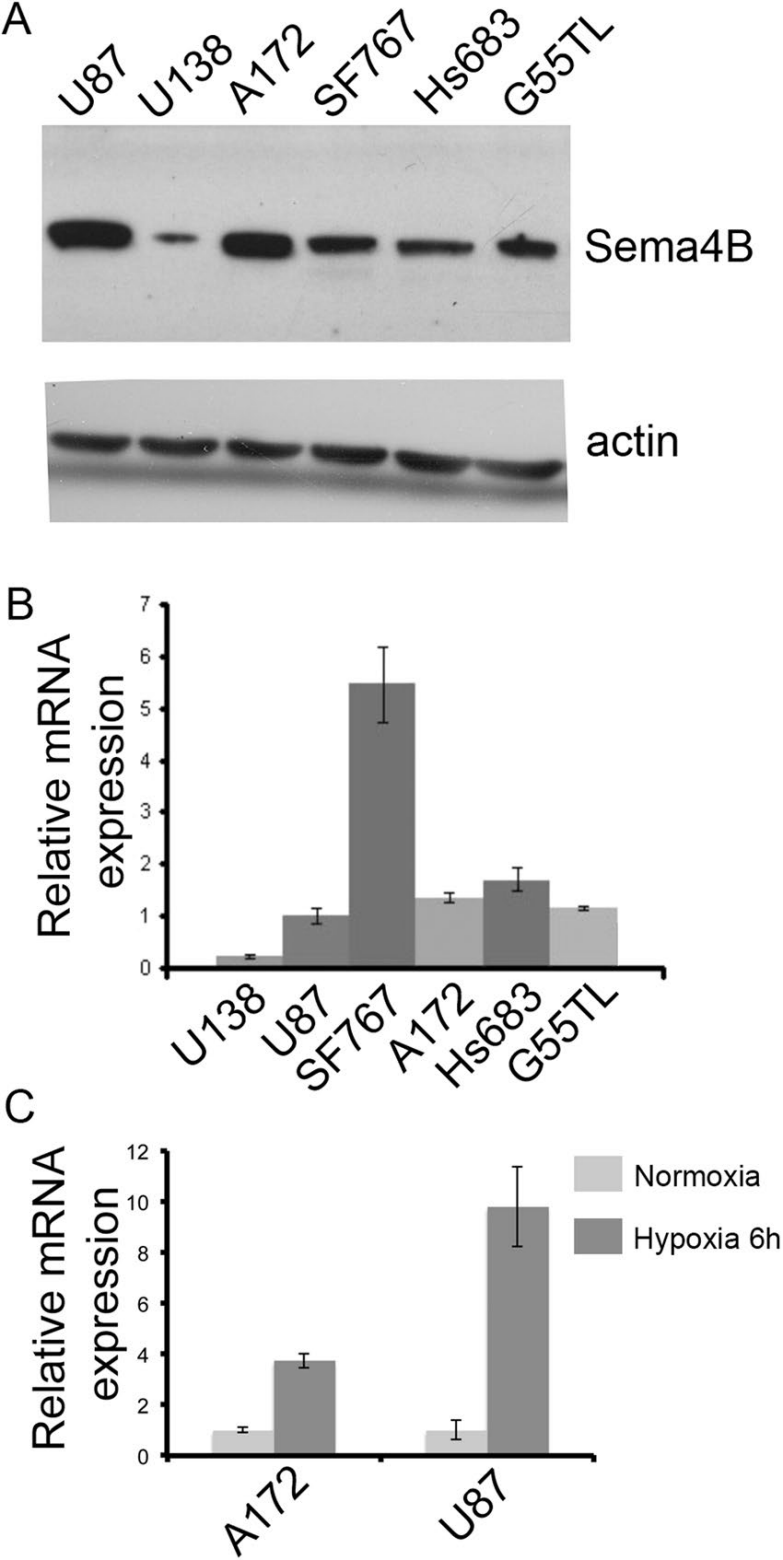
Expression of Sema4B in glioma cell lines. (**A**) Representative image of a western blot analysis of human glioma cell line extracts reveals expression of Sema4B in all cell lines tested. Actin was used as a loading control. The full-length blots are presented in Supplementary Fig. 3. (**B**) qPCR analysis of Sema4B mRNA in different glioma cell lines. Data are presented as mean ± s.e.m. (**C**) qPCR analysis of Sema4B mRNA under conditions of hypoxia. U87-MG and A127 were exposed to 1% or 21% oxygen for 6 h. Data are presented as mean ± s.e.m. The results in (**A**–**C**) are representative of three independent repetitions.

To begin testing the role of Sema4B in gliomas, we examined the expression of Sema4B in different glioma lines. Indeed, Sema4B protein and mRNA are expressed by all glioma cell lines tested (Fig. 1A,B), although these lines express Sema4B at different levels. Next, we tested whether Sema4B expression is regulated by hypoxia, as it is a predominant feature in GBM and its microenvironment^21^, and since Sema4B has been shown to be regulated by hypoxia-inducible factor 1^22^. Two lines of glioma cells (U87-MG and A172) were tested; in both cases Sema4B was up-regulated in response to hypoxia (Fig. 1C).

**Figure 2:**
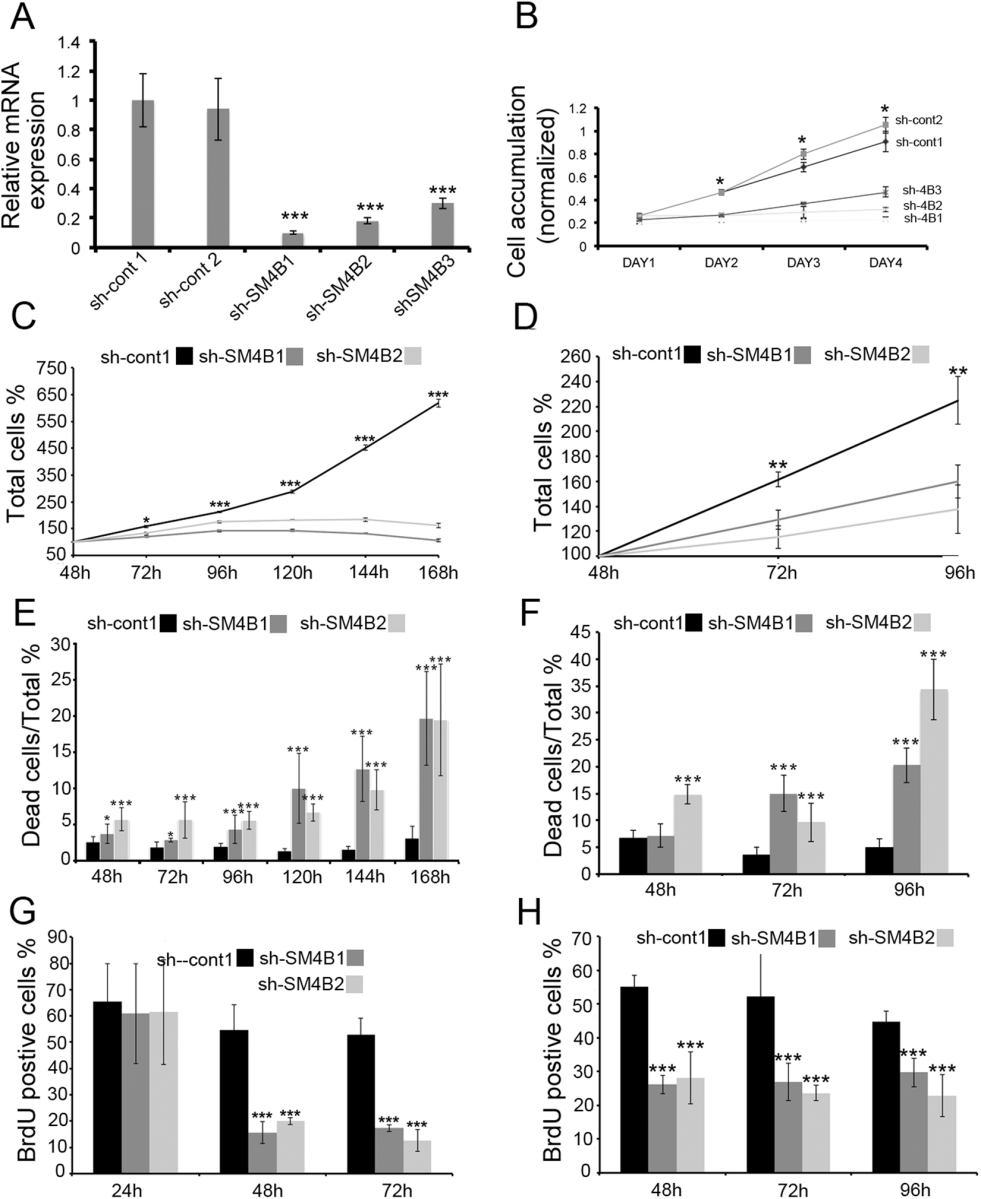
Knockdown of Sema4B inhibits proliferation and induces cell death. (**A**) qPCR results show that knockdown of endogenous Sema4B by three different shRNA sequences reduces the expression of this gene in U87-MG cells (data are presented as mean ± s.e.m., Mann–Whitney U test, n = 3). (**B**) XTT assay was used to evaluate U87-GM cell number at 1, 2, 3 and 4 days after plating. Each group of cells was treated with lentivirus expressing shRNA targeting Sema4B or control. The cells were plated for XTT assay 48 h after infection (data are presented as medians with range). P values were calculated with one-tailed Wilcoxon Signed Ranks Test (n = 5). (**C**–**H**) Effect of sh-cont and two sh-Sema4B were tested on U87-MG (**C**,**E**,**G**) or G55TL (**D**,**F**,**H**). (**C**,**D**) The same fields were monitored from 48–168 h (**C**) or 48–96 h (**D**) after infection (data are presented as mean ± s.e.m., n = 3). The p values were calculated with the Chi-square Fisher’s Exact test. (**G**,**H**) Glioma cell death was monitored using live/dead assay from 48 h up to 168 h (**E**) or up to 96 h (**F**). Data represent the means ± s.e.m. of three experiments (n = 3); p values were calculated with Chi-square Fisher’s Exact test. (**G**,**H**) Cell proliferation was monitored by adding a BrdU pulse 2 h before fixation. Proliferation was tested at 24–72 h (**G**) or 48–96 h (**H**). Percentage of BrdU-positive cells of the total DAPI-positive cells in each field is presented. Data represent the means ± s.e.m. of three experiments (n = 3); p values were calculated with Chi-square Fisher’s Exact test. *P < 0.05, **p < 0.001 ***p < 0.001.

To study the biological effect of Sema4B in glioma cells, we tested the effect of its depletion using the well-established shRNA approach. As a first step, we used three different shRNA sequences from the RNAi Consortium shRNA Library (MISSION shRNA library) and two control vectors (one empty vector and one scrambled shRNA). Each virus was titered and used at a concentration of about one infecting unit/cell to reduce non specific effects. Using qPCR, we monitored the level of Sema4B expression in U87-MG cells at different time points after infection. At 24 h after infection, there was no change in Sema4B expression. By 48 h there was a significant reduction in expression (Supplementary Fig. 1), and by 72 h after infection there was a consistent reduction of about 70–90% in Sema4B expression, depending on the sequence used (Fig. 2A). To test the effects of the shRNA treatments on cell proliferation/survival, we started with U87-MG and monitored their effect using an XTT assay. In all three shRNA vectors targeted towards Sema4B, U87-MG cell numbers were lower compared to control vectors (Fig. 2B). Interestingly, the effects detected by XTT in each shRNA correlated well with the effects of each sequence on the levels of Sema4B. Since both controls and all three specific shRNAs gave consistent results, we continued our experiments with one control and two specific shRNAs. To exclude the possibility that any effective mRNA expression results in non-specific effects on U87MG cell proliferation, we decided to target PlexinB2 by shRNA. All 3 PlexinB2 shRNAs reduced about 70–90% of PlexinB2 expression without affecting cell proliferation (Supplementary Fig. 2A,B).

To further characterize the effects of Sema4B depletion, we repeated the shRNA treatment with two glioma lines (U87-MG and G55TL, a glioma stem cell line) and monitored the number of cells for up to 168 h (in U87-MG) or up to 96 h (in G55TL). We followed the number of cells in ten specific fields for each treatment every 24 h using scanning stage methodology. In both cell lines there was a decrease in accumulation of cells over time (Fig. 2C,D). Since this result can represent a cell proliferation defect, we used BrdU labeling to follow proliferation rate. We used the same two glioma cell lines and in both cases we detected a significant reduction in cell proliferation 48 h after shRNA infection (Fig. 2E,F). The reduction in proliferation after 48 h correlated well with the expression of Sema4B, which was inhibited after 48 h. In addition to proliferation, the difference in cell number after shRNA treatment can also be explained by increased cell death. To test this point, we used a live/death assay. There was an increase in cell death in both cell lines tested, albeit with somewhat different kinetics. In U87-MG cells, death was detected in both shRNA after 96 h, and by 168 h there was about 20% cell death. Similar results were also seen with G55TL, with slightly more accelerated kinetics (Fig. 2G,H).

### Sema4B knockdown disturbs additional functions in glioma cells

**Figure 3:**
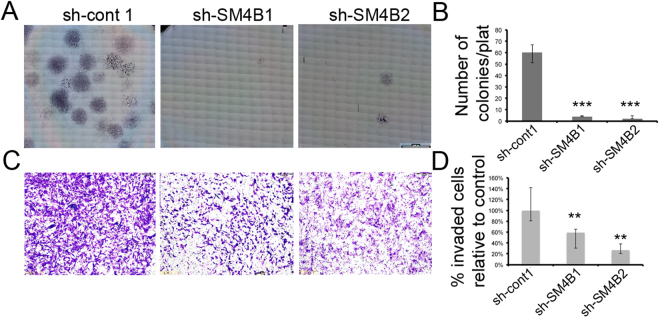
Knockdown of Sema4B reduces colony formation and cell invasion. (**A**,**B**) Colony formation assay experiments (n = 3) were performed for sh-cont and two shRNA targeting Sema4B. Forty-eight (48) h after treatment with the shRNA, 400 cells were seeded in each 6-well plate and stained with Giemsa after 18 days. (**A**) Example of the results after Giemsa staining. (**B**) Colonies were counted for each well; data represent the median of three experiments (the p values were calculated with the one-tailed Fisher’s Exact Test, n = 400; p < 3.148E-12). (**C**,**D**) Invasion assays using Boyden chamber transwell experiments were performed for sh-cont and two shRNAs targeting Sema4B (n = 3). (**C**) Invading cells were observed after 7 hours. (**D**) Six pictures were taken of each treatment and invaded cells were counted. Data represent the median number of counted cells relative to the control, with range (n = 6; p < 0.004). Data are presented as medians with range. The p values were calculated with the two-tailed Mann–Whitney Test.

To better understand the influence of Sema4B on glioma cells, we performed two additional assays: (1) the ability to form colonies in very high dilution; and (2) the ability to migrate. Following Sema4B knockdown, there were almost no U87-MG colonies detected, in sharp contrast to the control cells’ colonies (Fig. 3A,B). Sema4B knockdown also reduced the migration of cells in a Boyden migration assay, although the effects were not as dramatic as in the colony formation assay (Fig. 3C,D).

**Figure 4:**
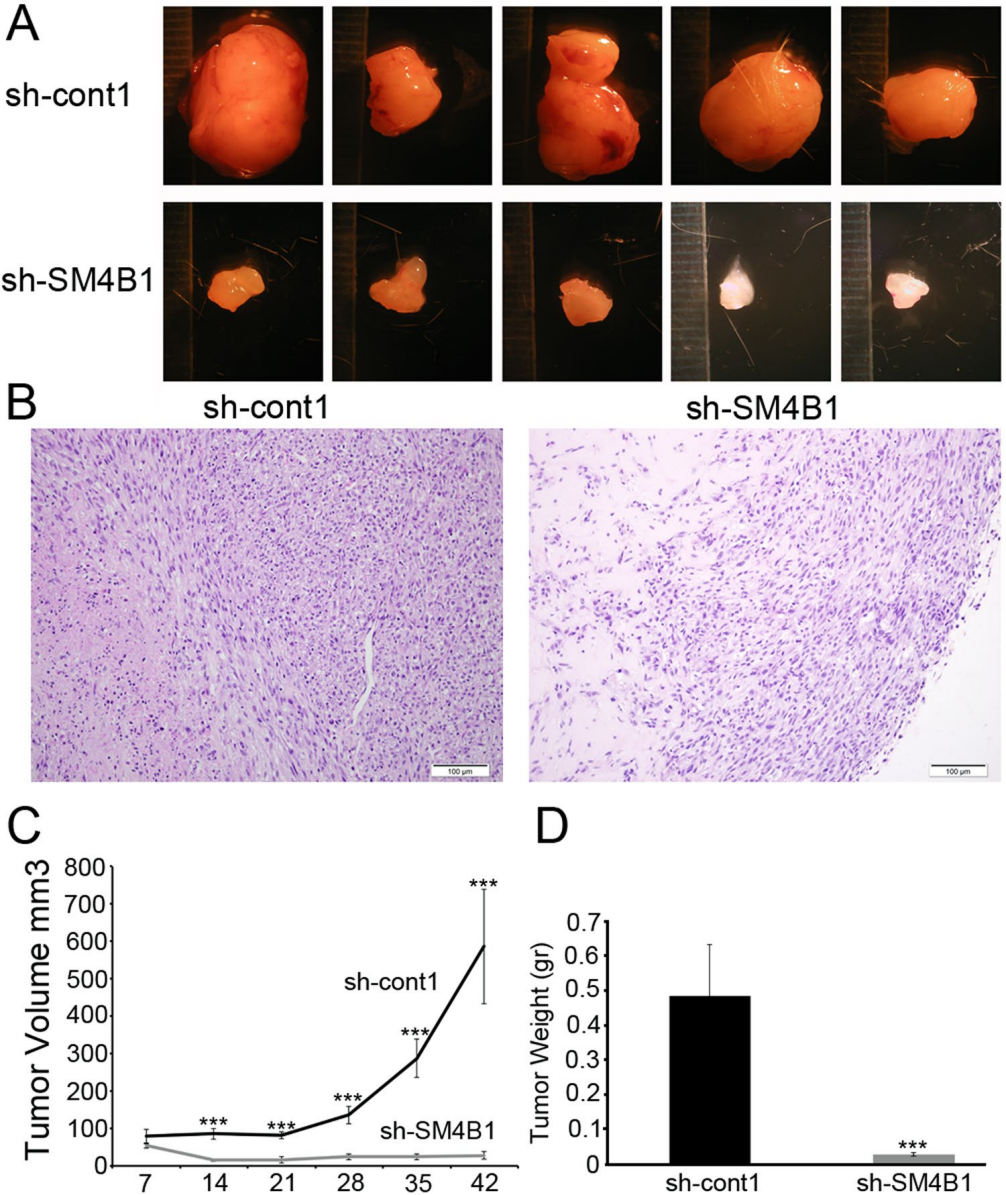
Knockdown of Sema4B reduces tumor formation *in vivo* using a xenograft model. (**A**–**D**) Two groups of U87-MG cells were infected with either shRNA lentivirus targeting Sema4B or control and selected for three days before the beginning of the experiment. Each group of cells was injected subcutaneously into nude mice. (**A**) Macroscopic appearance of xenografts at the end of treatment. (**B**) Section of xenografts stained with hematoxylin/eosin. Scale bar 100 μm. (**C**) Change of tumor volume during treatment period. (**D**) Tumor weight at the end of the treatment. Data represent the means of n = 5 ± s.e.m. The p values were calculated after the data were confirmed to fulfill the criteria with Mann–Whitney U Test: p < 0.005.

To test the impact on tumor formation, we used a xenograft model to begin validating the proliferative and survival effects of Sema4B *in vivo*. In this model we found that tumors are formed in both cell lines, although the size of the tumor volume and weight was markedly reduced in Sema4B knockdown cells (Fig. 4).

### Sema4B Knockdown and rescue attempts with mouse Sema4B

**Figure 5:**
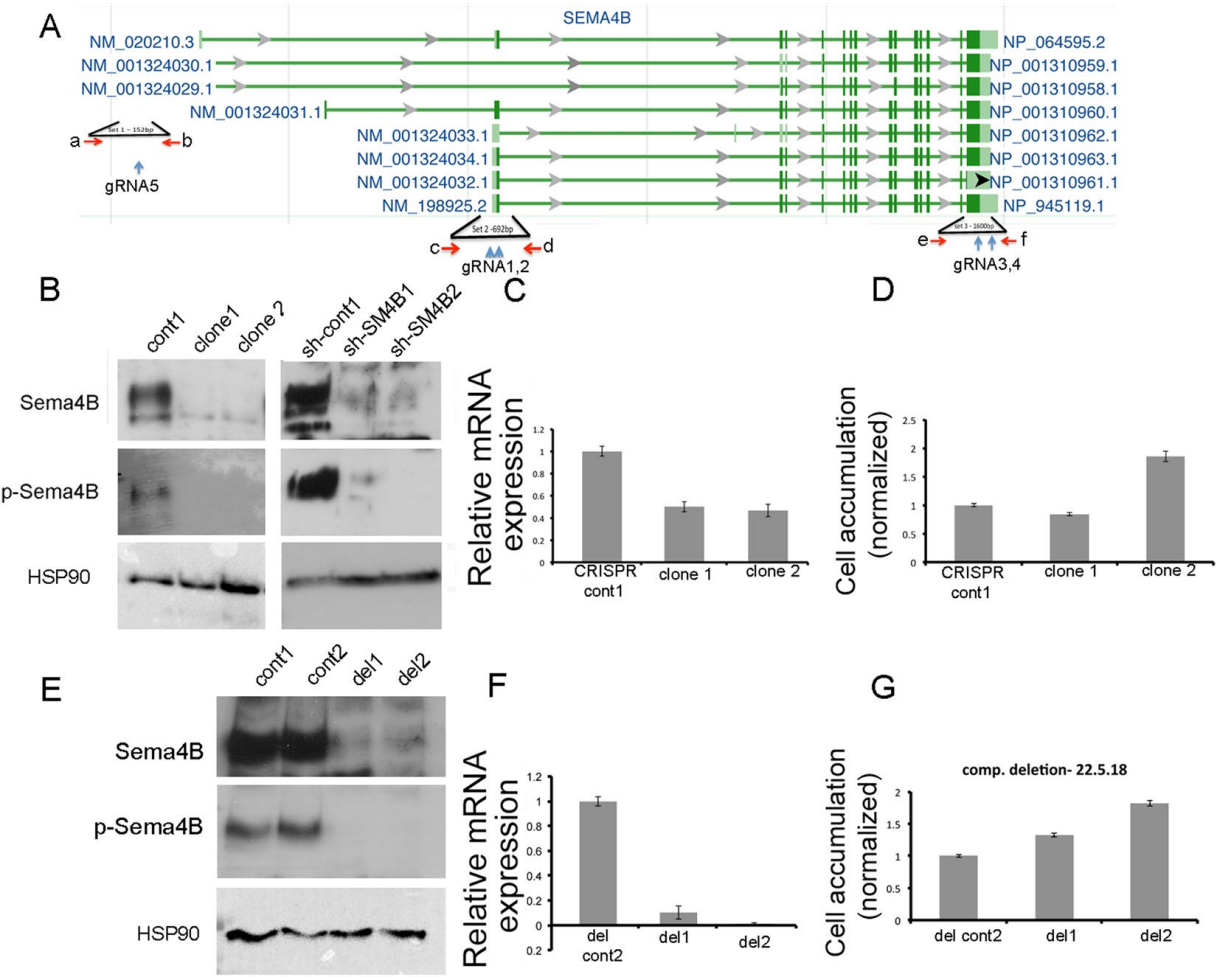
CRISPR-cas9 mediated mutations and deletion of almost the entire genomic locus of Sema4B do not effect proliferation. (**A**) Map of the genomic locus of Sema4B showing the potential alternative splicing, location of the gRNA targets and PCR targets used for the analysis of the genomic locus after CRISPR-cas9 mediated mutations or deletion. (**B**) Representative western blot of U87-GM cell pools treated with sh-cont1 or shRNA1 or 2. In addition protein extracted from cell clones generated by CRISPR-cas9 and cont1 gRNA (clone1), gRNA1 (clone 1) or gRNA 2 (clone 2) targeting the signal sequence of Sema4B are shown. The full-length blots are presented in Supplementary Fig. 4. (**C**) qPCR analysis of the same CRISPR cell clones demonstrate a variable effect on mRNA expression of individual clones. (**D**) Resazurin cell viability assay was used to evaluate the CRISPR clones in cell proliferation assay. Cells were tested one day after plating and four days in culture. The fluorescent read on day 4 was normalized to the read of the same cell line on day 1. No effects on proliferation are detected. (**E**) Representative western blot of U87-GM cell clones generated by two rounds of CRISPR-cas9 with two sets of gRNA in each round (the full-length blots are presented in Supplementary Fig. 4). One control line (cont2) and two deletion lines are shown (del1 and del2). Two cell clones were negative for oligo sets c,d and e,f (both were positive for fragment a,b) qPCR analysis of the same del1 and del2 cell clones show that mRNA levels of both clones are very low with del 2 almost undetectable. (**G**) Resazurin cell viability assay was used to evaluate del1 and del2 clones in cell proliferation assay. Cells were tested one day after plating and four days in culture. The number of cells on day 4 was normalized to the same cell line on day 1. No effects on proliferation are detected.

To validate the specificity of the effects of the shRNA on the glioma cells, we attempted to use a rescue strategy, in which we infected the same cells with both the mouse Sema4B cDNA and shRNA directed at the human Sema4B. We have previously successfully employed a similar strategy using this mouse Sema4B vector to rescue the function of Sema4B in null astrocytes^20^. We therefore assume this modified version of Sema4B generates functional proteins. To monitor the expression of the added Sema4B, we used a myc tag. The mouse Sema4B exhibited high expression levels, while the human Sema4B was repressed (as determined by qPCR). We used BrdU labeling to test the proliferation rate of the U87-MG cells expressing the mouse Sema4B but were unable to get consistent results. In some experiments we detected variable degrees of rescue while in others no rescue was detected (Supplementary Fig. 2). Various explanations might explain these results, however, it is worth noting that according to the NCBI (Gene ID 10509) the human Sema4B gene has 8 splice variants that result in different amino acid sequences especially at the N-terminus (see Fig. 5A) of the protein while the mouse cDNA has only four splice variants. One cannot ignore the possibility that a unique splice variant of the human gene is necessary for the activity of Sema4B in gliomas.

### Using CRISPR-Cas9 approach as an alternative approach to Sema4B function in glioma cells

Since we were not been able to get confirmation for the role of Sema4B using our rescue strategy, we chose an alternative approach to inhibit Sema4B, namely the CRISPR-Cas9 approach. For this, we started with 2 different gRNAs targeting the signal sequence of Sema4B. We isolated 2 clones (one for each gRNA) and tested RNA and protein expression for each line. In western blots of the two lines, the major band of the Sema4B protein was undetectable (Fig. 5B). However, a faint lower band was still detectable, and at this point we cannot rule out cross reactivity for another protein or a splice variant not targeted by our CRISPR construct (NM_001324033.1). As a control, we compared these cell lines to U87-MG cells treated with shRNA targeting Sema4B (Fig. 5B). The effects on RNA expression in the different CRISPR clones were also tested (Fig. 5C). We next tested the effects on glioma cell proliferation using the Resazurin cell viability assay. Surprisingly, there was no effect on cell proliferation (Fig. 5D). What mechanism can explain these differences between the two methods? First, it is theoretically possible that the mRNA itself has a function. Second, based on the NCBI gene data, 8 different splice variants were identified in the human Sema4B gene (Fig. 5A). All 3 shRNA sequences target all 8 alternative splice variants. In contrast, the two CRISPR-Cas9 gRNAs target only 6 splice variants (isoforms 2 and 3 are not targeted by the single CRISPR gRNA used). To exclude the possibility that the mRNA of Sema4B or one of these splice variants is responsible for the differences between the shRNA and CRISPR-Cas9 we decided to use another approach. For this method we used 4 gRNAs, with each set of 2 gRNAs targeting the 3′ and the 5′ of the Sema4B genome locus with the purpose of deleting most coding exons of this gene. For this we infected U87-MG cells already expressing Cas9, in two consecutive rounds a day apart. For the first round of infection we used gRNAs 4 and 5, and a day later the same cells were again infected, this time with gRNAs 1 and 3 (See Fig. 5A for location of each gRNA). These two infection cycles were designed to increase probability of a complete deletion event of the Sema4B locus. We then isolated individual cell clones and used 3 sets of primers to map the region of deletion (Fig. 5A, oligo sets a and b, c and d, &e and f). We identified two clones in which about 30,000 bp were excised, resulting in removal of most of the coding regions of Sema4B (both clones were positive for set A and B, and negative for both sets C and D, and E and F). To make sure Sema4B was completely deleted, we used both qPCR and western blot (Fig. 5D,E). At the protein level we did not see any positive signal, including the smaller band seen in the single CRISPR lines, suggesting that this is indeed a splice variant that exists in U87-MG cells. At the mRNA level, there appeared to be a very low level of expression, indicating these lines are not 100% pure cells (although we re-purified our clones, getting a pure line without a trace of Sema4B was impossible). Nevertheless, proliferation of both clones was a bit higher than the control lines (expressing the same levels of Cas9 and two sets of non-target gRNA sequences, Fig. 5F). Since CRISPR-Cas9 assisted deletion of the genomic locus resulted in no detectable protein and almost no mRNA, and yet the cells proliferated like control cells, we were able to exclude the possibility that splice variants, or mRNA levels are the cause for the differences between shRNA and CRISPR-Cas9 lines with respect to proliferation.

**Figure 6:**
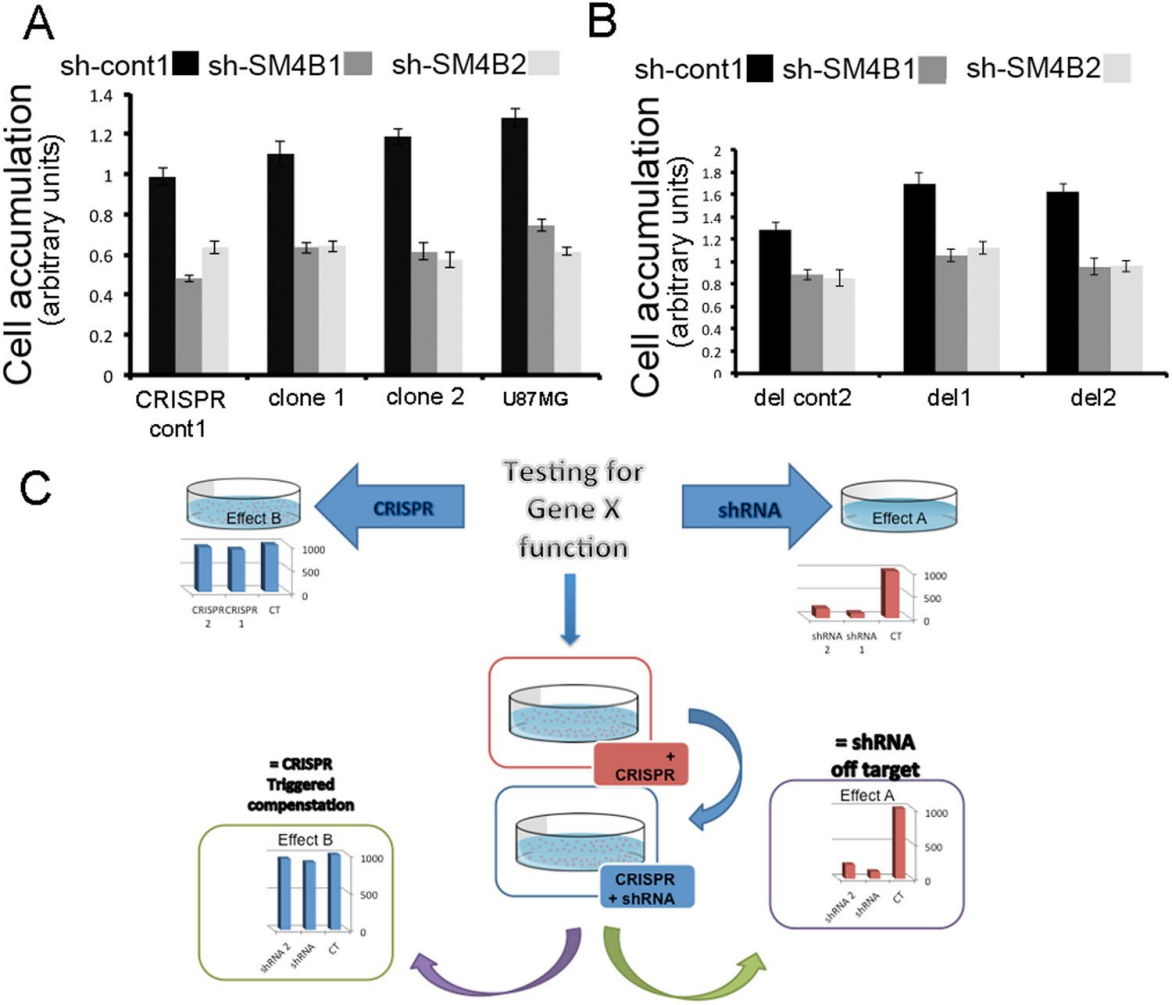
Combined shRNA over CRISPR/cas9 as a methodology to detect off targets and potential compensatory mechanism. (**A**–**C**) shRNA over CRISPR/cas9 cell lines. (**A**,**B**) Resazurin cell viability assay was used to evaluate cell number. CRISPR-cas9 clone 1 and 2 (mutations in signal sequence of Sema4B) and cont line 1 (**A**) or clones in which the genomic locus was deleted (**B**), del1, del2 and del-cont 2,) were subdivided each into three groups. Each group was infected with shRNA targeting Sema4B (sh-SM4B1, sh-SM4B2) or control (sh-cont). Note that in all four lines, whether or not they express Sema4B, proliferation was reduced by shRNA targeting Sema4B, thus demonstrating that the effects on proliferation are the result of an off target effect. Data in (**A**) and (**B**) represents the means of n = 3 independent repetitions ± s.e.m. (**C**) Flow chart demonstrating the steps in the methodology to differentiate between off target effects and compensatory mechanism. (1) To test the role of a gene X in a specific function we propose to compare the results of shRNA (or siRNA) with the effects of CRISPR-Cas9 targeting the same gene X. In cases in which shRNA has an effect while CRISPR-Cas9 does not, we propose to first mutate gene X using the CRISPR-Cas9 method, followed by shRNA to the same gene. There are two possible outcomes: (1) a result similar to the effects of shRNA = an off target effect; (2) a result similar to the effects of CRISPR-Cas9 = result represents a compensation mechanism triggered by the genetic mutation.

We were therefore left with two possibilities: off-target effects of all shRNA used or genetic compensation under conditions of a complete loss-of-function due to genetic mutation. To determine which was the case here, we combined both approaches in one experiment. We used single CRISPR clones 1 and 2, (Fig. 6A) or del clones 1 and 2 (complete deletion of the genomic locus of Sema4B). We then infected each CRISPR clone with three types of shRNAs, either sh-cont (control) or one of two sh-SM4Bs, after which we tested the glioma cell proliferation. Regardless of whether Sema4B was intact or lost within the glioma CRISPR clones (in control clones or deleted ones), all lines responded similarly when treated with sh-SM4Bs (one infecting unit/cell as determined by testing parental U87MG). A slightly weaker effect took place especially in the complete deletion trial (in del-cont and del 1 and 2). This is probably the result of a reduced infection ability of these lines, which underwent 3 previous viral infections. Consistent with this possibility, we see reduced knockdown in the del-cont line (Supplementary Fig. 4). Alternatively, this slightly weaker effect may represent a very modest compensatory response. However, since the reduced response is most pronounced in the control line, it most likely indicates that the effects of the sh-SM4B are the result of an off-target effect (Fig. 6A,B). Based on these results, we propose a general methodology to test gene function in cases in which inhibition by CRISPR-Cas9 has no effect, while RNA interference does have an effect (Fig. 6C). By using RNA interference on cells after genetic mutation in the same gene introduced by CRISPR-Cas9, it is possible to determine whether the inconsistency is the result of an off-target effect by RNA inhibition or a compensatory mechanism induced by the genetic mutation.

### Sema4B is needed for clonal formation

**Figure 7:**
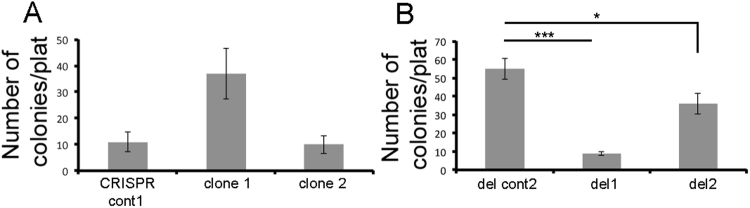
CRISPR-cas9 mediated deletion of genomic locus of Sema4B results in reduced clonal potential. (**A**,**B**) Colony formation assay experiments (n = 3) were performed for cell line mutated with single CRISPR ((**A**), clone 1, 2 and CRISPR cont 1) or cells with CRISPR assisted deletion of the genomic locus of Sema4B ((**B)**, del 1, del 2 or CRISPR control 2). 400 cells were seeded in each 6-well plate and stained with Giemsa after 18 days. Colonies were counted for each well; data represent the median of 9 plates in three experiments (the p values were calculated with the one-tailed Wilcoxon-Mann-Whitney Test * = 0.0328, *** = 0.0567668E-5).

Thus far we have shown that the effects of RNA inhibition on glioma proliferation is the result of an off target effect. Next, since shRNA inhibition of Sema4B also had strong effects in a clonal assay (Fig. 3A,B), we decided to test whether this is also a result of an off target effect. We used the same assays (as were used for the shRNA trials), first on the CRISPR single clones targeting the signal sequence of Sema4B (Fig. 7A). The results using these assays show no difference in comparison to the control lines. We also tried this assay with the two clones with complete deletion of the genomic locus of Sema4B (del clones 1 and 2). Surprisingly, in this assay we detected a reduction in clonality (Fig. 7B). The difference between the single CRISPR and the total deletion of Sema4B probably indicates that one of the splice variants not targeted by the first approach is the functional form of Sema4B in this system. Thus, we conclude that although the shRNA effects on proliferation are the result of an off target effect of shRNA, the reduced clonality of the cells, confirmed by CRISPR, probably indicates that certain Sema4B splice variants do have a role in some aspects of cancer properties, at least in U87-MG.

## Discussion

In this study, we used the well-established and widely used shRNA methodology for the testing of gene function. Although we used three different shRNAs and there was a linear correlation between the degree of gene inhibition and the biological phenotype, our results clearly demonstrate that the dramatic effects on cell proliferation are the result of off-target effects. Over the years, the understanding of the need to add rescue experiments has become more apparent. However, rescue experiments in which the repressed gene is reintroduced, entail some limitations, including the fact that overexpression of exogenous genes may result in non-specific effects and the fact that splice variants may make the rescue experiments more challenging. Thus, the case of Sema4B in glioma as presented here should be considered as a warning to the scientific community to critically read publications that rely mostly on RNA interfering strategies. On the other hand, our experiments also demonstrate the caution in which the CRISPR method should be considered, as its inhibition is at the DNA level and therefore it is possible that certain splice variants will not be affected.

The use of genetic mutation to study gene function is a powerful technology. Nevertheless, over the years there have been numerous cases of gene inactivation which didn’t result with the predicted phenotype, based on known functions of this gene and its expression^23^. One explanation for the lack of phenotype is redundancy, which can be at the level of an individual gene, such as that between isoenzymes, which are generated by gene duplication. This is the case for SRC family members Fgr and Hck in erythrocyte K/Cl cotransport^24^. Redundancy can also occur at the system level, due to distributed properties of networks. For example, glucose-6-phosphate dehydrogenase and D-ribulose-5-phosphate 3-epimerase catalyze distinct reactions and are located in alternative pentose phosphate pathways in yeast; simultaneous removal of the two enzymes is lethal, although individual removal of either enzyme is not^25^. An important consequence of genetic redundancy is robustness against genetic perturbations such as deleterious mutations. It is challenging to estimate to what degree there are null mutant genes without observable phenotype in complex organisms. Some indication of the scale of the problem can be estimated from data obtained in single-cell organisms such as yeast. Use of a systematic approach to generate loss-of-function mutations showed that about 40–60% of the mutants had no detectable phenotype (growth defects, shape and size abnormalities) in the assays used^26^. Is the genetic redundancy specific to loss-of-function mutations or do other methods such as RNA interference also activate such mechanisms? A recent report in zebrafish showed that, at least in the case of the egfl7 gene, compensation is triggered by genetic mutation but not by more modest repression of gene expression using anti-sense morpholino^15^. It remains to be seen how abundant this phenomenon is.

Gene knockdown by siRNA is, without a doubt, prone to off-target effects^1,27^. Thus, rescue experiments are important, though in cases of multiple splicing variants might complicate such an experimental approach. Also, as was demonstrated recently, complete loss of gene function by genomic mutations may induce a compensatory mechanism that will prevent detection of the function of a gene^15^. Therefore, in cases in which shRNA or siRNA affect cellular function while CRISPR-cas9 does not, we propose a combined shRNA (or siRNA) over CRISPR-cas9 approach. Such an approach maybe a useful way to differentiate, under certain conditions, between an off-target effect and a potential compensatory mechanism.

The case of Sema4B function in glioma presented here is complex. Clearly this gene has no role in glioma proliferation (at least not in U87-MG cells). We can further conclude that the well characterized forms of this gene that includes the signal sequence and the complete sema domain of this gene also have no detectable role in glioma. Nonetheless, it seems that one or two splicing variants, that do not contain the beginning of the gene (the signal sequence and the start of the sema domain), do have an important function in the ability of cells to grow in low density. All the same, the exact role of Sema4B in glioma biology will need further investigation.

## Methods

Antibodies: anti-Sema4B GeneTex cat #GTX121035- this antibody is directed to the N-terminus of Sema4B and according to the manufacturer expected to recognize 7 out of 8 possible splice variants (see Fig. 5) but will not recognize isoform 2 (NP_001310958.1). Anti-p-Sema4B(Ser825) at the C-terminus of Sema4B, cat #5622 CST and,anti-HSP90 BD Biosciences #610418, anti-actin CST #4967.

We also tested the anti-Sema4B from CST (#13771) that worked very well on mouse samples but was non-specific in U87-MG cells. The Abcam ab81130, Sigma HPA13372 and Aviva antibody cat # ARP49485 antibodies were not specific.

### Cell Culture

Human glioblastoma cell line U87-MG and A172 are from the ATCC (Virginia, USA), and the cell line G55TL primary glioblastoma cell cultures^28^ provided by Prof. Till Acker from the Institute of Neuropathology Medicine, University Giessen, Germany.

### Animals

NOD/SCID mice were obtained from Harlan Laboratories. The Ethics Committee of the Hebrew University approved all animal experimental protocols in this study and these protocols were strictly followed.

### Immunoblots

Cells were harvested in lysis buffer (1% NP40, 0.5% sodium deoxycholate, 0.1% SDS, 150 mM NaCl, 10 mM buffered phosphate pH 7.2, 2 mM EDTA, 50 mM NaF, 0.2 mM orthovanadate and protease inhibitor cocktail). Cells were collected with a cell scraper, passed six times through a pipette tip, vortexed, and incubated on ice for 15 min. The lysates were then centrifuged at 20,000 × g for 15 min and the pellets discarded. Protein concentration of each sample was determined using Bradford reagent (Sigma). Samples were boiled in 1 × SDS sample buffer, separated by SDS-10% polyacrylamide gel electrophoresis (PAGE) and blotted onto PVDF membranes (Millipore). The membranes were incubated in 5% fat-free milk in TBST (10 mMTris-HCL pH 7.4, 150 mM NaCl, 0.1% Tween 20) for 1 h and then 5% BSA in TBST containing various dilutions of primary antibodies for 18 h at 4 °C. The membranes were washed three times with TBST for 5 min each before and after incubation with secondary antibody. The proteins were detected with an appropriate secondary antibody (1 h, RT) coupled with horseradish peroxidase-conjugated goat anti-rabbit or anti-mouse antibody and visualized by chemiluminescence according to the manufacturer’s instructions (West Pico, Pierce).

### Sema4B knockdown

For Sema4B knockdown experiments we used MISSION® shRNA lentiviral vectors (pLKO.1) which includes puromycin selection marker: TRCN0000061193 (sh-SM4B1), TRCN0000061194 (sh-SM4B2) and TRCN0000061195 (sh-SM4B3). For control we used empty vector (sh-cont1) and TRCN0000112294 (sh-cont2) all purchased from Sigma Aldrich. For PlexinB2 knockdown experiments we used MISSION® shRNA lentivirus vectors (pLKO.1), TRCN0000300489 (sh-B2-1), TRCN0000300549 (sh-B2-2), TRCN0000381500 (sh-B2-3).

Lentiviral infections: Each set of lentivirus was carefully evaluated for its titer. A day later puromycin selection was applied and a day after selection the number of cells in each well was counted. From these results we estimated the transducing units/ml for each set of shRNA prep.

Each shRNA sequence was then tested for the degree of knockdown using multiplicity of infection (1, 5 and 10). For all shRNA sequences we concluded that one infection unit/cell is sufficient to achieve reduction of Sema4B mRNA levels by at least 70%. In all following experiments we used an estimate of one infection unit/cell in all experiments.

### CRISPR-Cas9

Templates for gRNA were cloned into lentiCRISPR v2 (Addgene Plasmid #52961).

Single CRISPR: For this experiment we used gRNA sequences that targeted the first coding exon downstream to the first

ATG start site: The sequences are:

SM4B gRNA1:

5′-CGCACCGCGATGGGCCTG

5′-CAGGCCCATCGCGGTGCGC

SM4B gRNA2:

5′-GCTGGCTCGCCGCCCCAT

5′ATGGGGCGGCGAGCCAGCC

For control we used two gRNA without a target sequence in the human genome. The sequences are:

cont gRNA1

5′-AGCCGCTCCGCTGTCCTG

5′-CAGGACAGCGGAGCGGCTC

cont gRNA2

5′-CTGCGGACGACGACTACG

5′-CGTAGTCGTCGTCCGCAGC

gRNA expression with lentiviral infections: As in the case of shRNA we estimated the transducing units/ml based on the puromycin selection. In these experiments we used an estimate of one infection unit/cell.

Using sets of gRNA to remove genomic fragments:

In experiments in which two or more different gRNAs were used, the gRNAs were cloned into vectors without Cas9. In these experiments we used the following strategy: In step one, we established a sub line expressing cas9 without gRNA. This line was then treated with different gRNA sequences. This step was carried out in order to make sure all cell lines generated with the different gRNA sequences express the same levels of Cas9. We then did two cycles of infections with gRNAs 4 and 5 and a second round with gRNAs 1 and 3 (see Fig. 5A). For control lines we did two rounds of infections with control gRNAs 1 and 2. From each gRNA or gRNA set treated cells, individual clones were isolated and analyzed.

gRNA sets:

gRNA3

GTGAGAGCTGACTTCCAG

CTGGAAGTCAGCTCTCAC

gRNA4

GTATCCCCAGTGTGCCCC

GGGGCACACTGGGGATAC

gRNA5

AGGCAAGTGAGAGAGAGG

CCTCTCTCTCACTTGCCT

To test whether deletion of genomic locus occurred we first used PCR to test whether our clones were missing the genomic region of Sema4B. For this we used the following sets of oligos (a–f, position of oligos indicate in Fig. 5A):

Genomic set 1: a- Fw GCATTTATCTCGGGTGGAGA,

b-Rev CTGTGATGGGAAATGCAATG

Genomic set 2: cFw CCCACTTGACCCTGTTTCC

d,Rv AAGCAGTGGCCTCCCTCTAC

Genomic set 3:eFw TCACTAGAGGAGGGCTTCCA

fRv GAGGACCAGGGTGCAGTTAG. We used oligos c-f to amplify the DNA of clones del1 and del2. We were able to get a PCR fragment of about 1000 bp only in the case of clone del1. Using sequencing we were able to verify that about 30,000 bp were deleted from the genome of this line. We were not been able to get a PCR product in the case of clone del2, possibly because the deleted fragment was too big to amplify.

### Live cell count

Cells from cell lines U87-MG and G55TL were seeded on 6-well plates, 100 K cells per well. We photographed the same fields every 24 h after infection and selection. Cells were counted for every treatment and time period. Percentages of cells were determined according to the number of cells in the picture at time zero.

### Live/Dead assay

In order to assess the percentage of live and dead cells, we used ethidium homodimer (Sigma 46043) and 0.4 μl Calcein AM (Sigma C1359-100 μl). Four random fields were photographed in every well after 30 min with a computerized photosystem (Image Pro Plus software, camera Sensicam 12 Bit Cooled Imagine mounted on a microscope system Axiovert200, FITC-Live, Cy3-Dead). Cells were counted for every treatment and time period. Percentages of dead cells were determined according to the number of total cells in the picture at every time point.

### qPCR analysis

RNA was extracted using Quick-RNA MiniPrep (ZYMO R1055). Total RNA (500 ng, as determined by Nanodrop, purity 260/280 above 1.9) in a total volume of 20 μL was reverse-transcribed with the ImProm-II™ reverse transcriptase cDNA synthesis kit (Promega, Madison, WI) according to the manufacturer’s instructions. The resulting cDNA reaction mix was then diluted 20 times in double-distilled water. Real-time quantitative PCR (qPCR) was performed with the SYBR Green mix (Roche) according to the manufacturer’s instructions. The specific primers were as follows:

Human Sema4B primers (recognize all 8 splice variant):

Fw -pair 1 GGCCCTCTTTGCACTCAGTA

Rv-pair 1 TGTTTCTTCTCTGCGTCTGC

Fw -pair 2 GGCGAGCTCTACACTGGAAC

Rv - pair 2 GTAGGCTGAGGCCACAAAAG

Human GAPDH Fw: 5′-TCG ACA GTC AGC CGC ATC TTC-3′

Human GAPDH Rv: 5′-AAC AAA TCC GTT GAC TCC GAC-3′

Human HPRT Fw: 5′-ACTGGCAAAACAATGCAGACTTT-3′

Human HPRT Rv: 5′-GGTCCTTTTCACCAGCAAGCT-3′.

### Xenograft assay

The cells were mixed with Matrigel (BD) 96 h afterSema4B knockdown and injected subcutaneously into NOD/SCID mice using a 0.3 mm syringe. Tumor diameters were measured with digital calipers, and the tumor volume in mm^3^ was calculated by the formula: Volume = (width)^2^ × length/2 The measurements started a week after injection, by caliper.

### XTT assay

Two thousand U87 cells were seeded onto 96-well plates. As per manufacturer’s instructions, 50 µl of the XTT reagent and 1 µl of the XTT activator were added to each well 24 h later and the plates were incubated for 3 h. The plates were then read in an ELISA machine at 492 nm. This procedure was carried out every 24 h as required for the experiment.

### Resazurin cell viability assay

Three thousand U87 cells were seeded onto 96-well plates. A day later Resazurin solution (10% of cell culture volume) was added to the plate for two hours. The cell medium was then collected and fluorescence was tested. Fresh mediums were then added to each well and the cells were grown for two additional days. At day 3 the Resazurin solution was again added to the same plate for two hours. For each well the fluorescent read at day 3 was normalized by dividing the fluorescent read at day 3 with the one measured at day 0. The gain used to read the samples was automatically selected to be optimal to each read and thus the comparison between the two reads is relative (not absolute growth values).

### Invasion assay

An invasion assay was performed using Transwell chambers. Nuclepore™ Track-Etched Membranes Track (WhatmanWHA150446) were coated with Matrigel (1:20) in SFM. The bottom chamber contained MEM supplemented with 10% FBS. The cells were allowed to invade for 7 hours at 37 °C, at which time the Matrigel and cells that were associated with the top surfaces of the membranes were removed with cotton swabs. Cells that penetrated through the Matrigel to the underside surfaces of the membranes were fixed and stained with Diff-Quick stain set (SiemensB4132-1A).

### Colony formation assay

For each treatment, cells were seeded in triplicate, 400 cells per well, in 6-well culture plates. After 18 days of incubation the cells were fixed with 100% ethanol for 10 minutes, and then stained with Giemsa (Sigma GS500) for 30 minutes. The colonies were counted.

### Hypoxia

Cells were plated on either 10 cm or 6 cm plates overnight. The next day the plates were incubated in a closed chamber with an anaerobic pack that absorbs oxygen and leaves 1% oxygen, or at 21% oxygen levels for 6 hours, after which RNA was extracted.

### Statistical analysis

Real-time PCR, xenograft tumor volume and tumor weight: Data are presented as means ± s.e.m. The p values were calculated with the Mann–Whitney U test after the data was confirmed as fulfilling the criteria. Cell count, BrdU, live/dead assay: Data are presented as means ± s.e.m. The p values were calculated with the Chi-Square Fisher’s Exact test. Symbols are as follows: *P < 0.05, **P < 0.001, ***P < 0.0001.

## Electronic supplementary material

Supplementary info and figures
