# Supplementary material for: Combined shRNA over CRISPR/cas9 as a methodology to detect off-target effects and a potential compensatory mechanism
Source: Sci Rep. 2018 Jan 8;8:93. doi: 10.1038/s41598-017-18551-z (PMC5758708; doi:10.1038/s41598-017-18551-z)
Supplement: Supplementary file 1 — Supplementary info and figures [file 41598_2017_18551_MOESM1_ESM.pdf]

Supplementary information

Title:

Combined shRNA over CRISPR/cas9 as a methodology to detect off-target effects and a potential compensatory mechanism

List all authors:

LiatPeretz*, ElazarBesser*, RenanaHajbi*, NataniaCasden*, Dan Ziv, NechamaKronenberg, Liat Ben Gigi, SaharSweetat, SalehKhawaled#, Rami Aqeilan# and Oded Behar

Corresponding author: Oded Behar

email: odedb@ekmd.huji.ac.il

Supplementary Fig 1: Time dependent mRNA reduction following Knockdown of Sema4B

qPCR results show that knockdown of endogenous Sema4B by two different shRNA sequences reduces the expression of this gene in U87-MG cells after 48h from the time of infection (data are presented as mean ± s.e.m., Mann–Whitney U test, n=3).

Supplementary Fig 2 - Knockdown of PlexinB2 does not inhibit proliferation and has mild effects on colony formation

(A) qPCR results show that knockdown of endogenous PlexinB2 by three different shRNA sequences reduces the expression of this gene in U87-

MG cells (data are presented as mean ± s.e.m.). (B) XTT assay was used to evaluate U87-GM cell number at 1, 2, 3 and 4 days after plating. Each group of cells was treated with lentivirus expressing shRNA targeting PlexinB2 or control. The cells were plated for XTT assay 48 h after infection (data are presented as medians with range). C) Colony formation assay experiments (n=3) were performed for sh-cont and 3 shRNA targeting PlexinB2. Forty-eight (48) h after treatment with the shRNA, 400 cells were seeded in each 6-well plate and stained with Giemsa after 18 days. Colonies were counted for each well; data represent the median of three experiments.

Supplementary Fig 3: Rescue with the Sema4B expression of shRNA knockdown resulted in inconsistent results.

U87-MG cells were infected with mouse Sema4B-myc or empty vector and after a day were selected with blasticidin. A day later the cells were re-infected with shRNA-cont, shRNA-SM4B1, or shRNA-SM4B2. A day later all cells were selected with puromycin and blasticidin. 72h after the infection with shRNA vectors, cell proliferation was tested and monitored by adding a BrdU pulse 2h before fixation. Percentage of BrdU-positive cells out of total DAPI positive cells in each field is presented. (In each experiment both Sema4B knockdown was verified as well as the expression of the myc tag). A. Data represent one (out of 3) experiments that show a degree of rescue. B. One (out of 4) experiments without any rescue.

Supplementary Fig 4: qPCR of Sema4B in del-cont line. qPCR results show that knockdown of endogenous Sema4B by two different shRNA sequences reduces the expression of this gene in U87-MG cells. However, knockdown is less effective than in parental U87MG cells (see figure 2A for comparison), probably as a result of reduced sensitivity of this sub-line to infection by the shSema4B lentiviruses).

Supplementary Fig 5: The full length of the gels presented in figure 1a is shown. These specific gels were generated with the same anti-Sema4B and anti-pSema4B used to generate figure 5, however they are both from different lots (the second lot is less clean but as can be seen in figure 5, the Sema4B protein is recognizable).

Supplementary Fig 6: The full length of the gels presented in figure 5B and E are shown. A. Isolated cell clones mutated by CRISPER-Cas9 targeting the signal sequence of Sema4B. The membranes were tested with anti-Sema4B, stripped and re-blotted with anti-pSema4B and finally with anti HSP90. B, C. cell pools treated with control or shRNA targeting Sema4B. Two different membranes are presented, one with anti Sema4B and one with p-Sema4B. The membranes were stripped and re-blotted with anti-HSP90. D. two control lines and two clones with complete deletion of most of the Sema4B are presented. The membranes were blotted with anti-Sema4B, stripped and re-blotted for anti-p-Sema4B and finally with anti-HSP90. Arrows mark

non-specific bands. Note that shRNA treatment reduces some non-specific bands identified by the anti-Sema4B antibody.

Supplementary Fig 1: Time dependent mRNA reduction following Knockdown of Sema4B

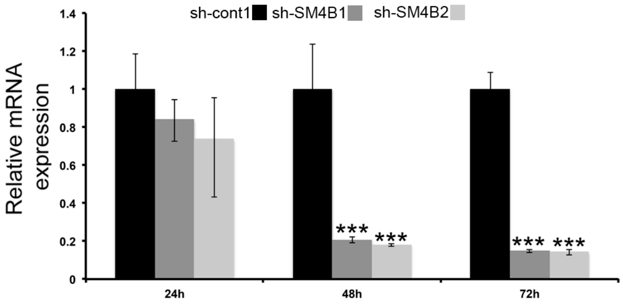

Supplementary Fig 2: Knockdown of PlexinB2 does not inhibit proliferation and has mild effects on colony formation

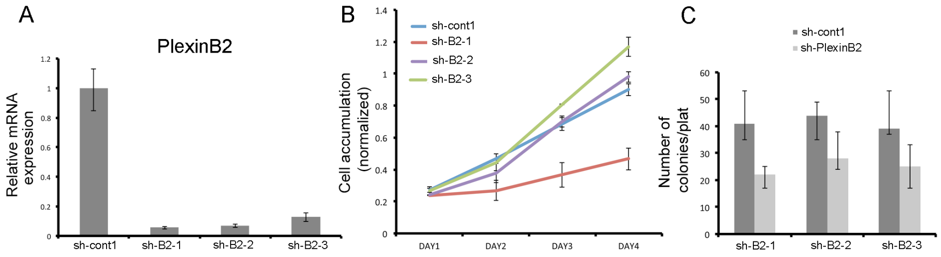

Supplementary Fig3: Rescue with the Sema4B expression of shRNA knockdown resulted in inconsistent results

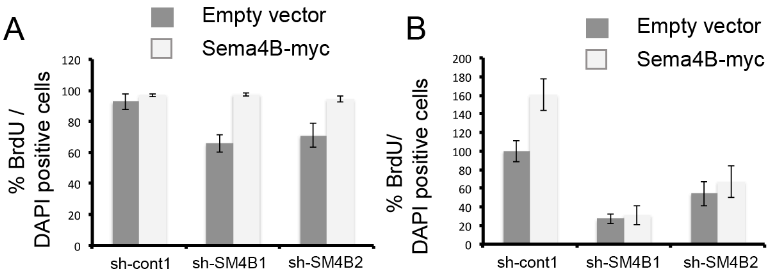

Supplementary Fig4: qPCR of Sema4B in del-cont line

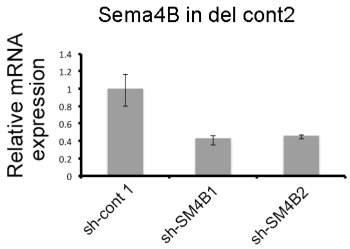

Supplementary Fig5: The full length of the gels presented in figure 1a

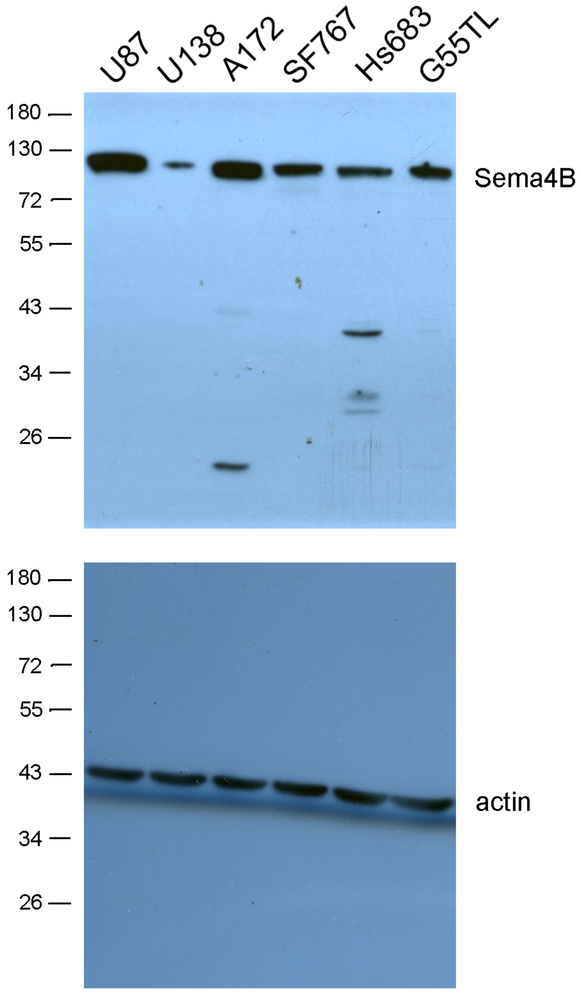

Supplementary Fig 6: The full length of the gels presented in figure 5B and E

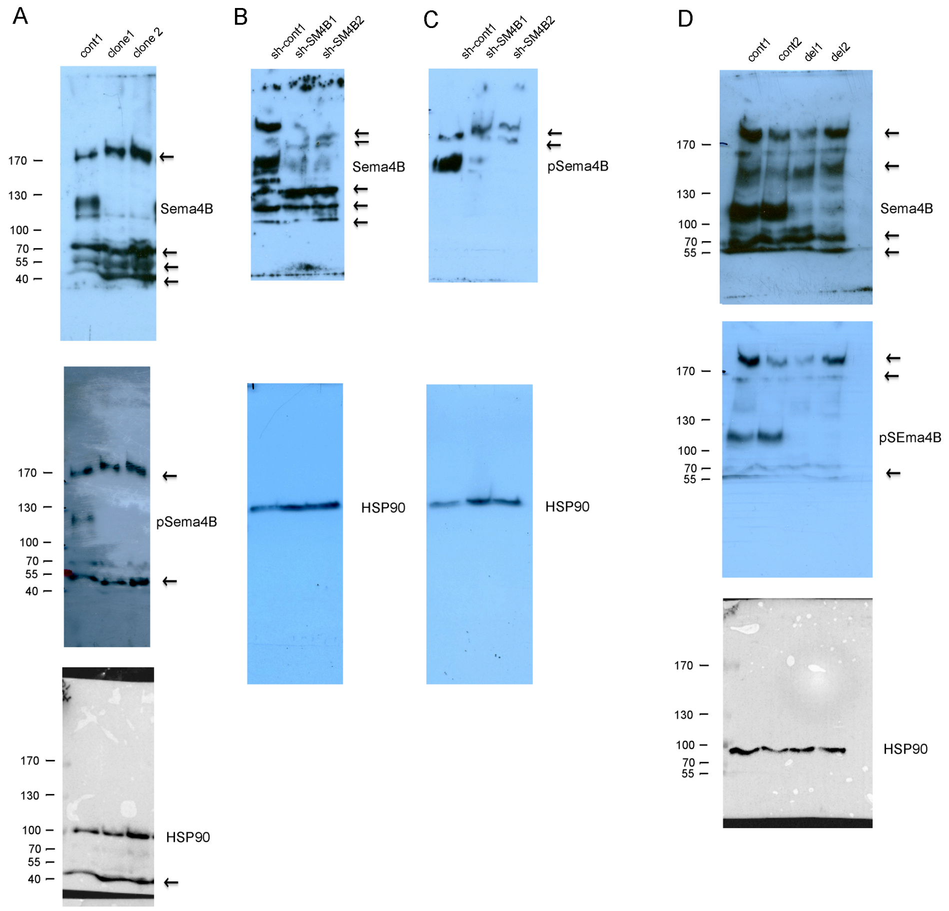

A
cont1
clone1
clone 2
170
130
100
70
55
40
Sema4B
pSema4B
HSP90
B
sh-cont1
sh-SM4B1
sh-SM4B2
Sema4B
HSP90
C
sh-cont1
sh-SM4B1
sh-SM4B2
pSema4B
HSP90
D
cont1
cont2
del1
del2
Sema4B
pSEma4B
HSP90
